# Commensals and Foodborne Pathogens can Arbitrate Epithelial-carcinogenesis

**DOI:** 10.9734/BMRJ/2016/26690

**Published:** 2016-06-10

**Authors:** Yvon Woappi, Om V. Singh

**Affiliations:** 1 Division of Biological and Health Sciences, University of Pittsburgh, Bradford, PA-16701, USA.

**Keywords:** Foodborne pathogens, carcinogenesis, inflammatory disease, commensal bacteria, toxins, immunity

## Abstract

Major shifts in intestinal commensal bacteria often result in changes in CD4^+^ T lymphocyte populations, leading to an influx of Th17 cells, chronic inflammation, and eventually cancer. Consequently, the inappropriate propagation of certain commensal species in the gut has been associated with mucosal inflammatory diseases and cancer development. Recent experiments investigating the relationships between food-borne pathogens, enteric bacteria, and cancer have exposed the ability of certain bacterial species to significantly reduce tumor size and tumor progression in mice. In similar studies, pro-inflammatory Th17 and Th1 cells were at times found present along with anti-inflammatory Treg populations in the intestinal mucosa. This antitumor response was mediated by a balanced production of pro- and anti-inflammatory cytokines, resulting in a controlled threshold of mucosal immunity largely moderated by CD4^+^ T lymphocyte populations, through a dendritic cell-dependent pathway. These findings provide new evidence that certain species of bacteria can help manage subcutaneous tumor development by calibrating mucosal and, in some instances, systemic thresholds of innate and adaptive immunity.

## 1. INTRODUCTION

The role of commensal bacteria and food-borne pathogens is now being recognized as a major driving factor in the development of many types of human cancers. In recent years, food-borne pathogens such as *Salmonella* spp. and *Escherichia coli* have been demonstrated to induce genomic mutations through the secretion of carcinogenic endotoxins and cytolethal distending toxins (CDT) harmful to mammalian cells [1–3]. And further study demonstrated that such strains, independent of immune cells, could successfully transform mammalian cells *in vitro*, thus highlighting microbials’ highly tumor-promoting characteristics [1,2]. Still, despite their transformed status, the cells required further genomic aberrations before becoming malignant. *In vitro* this is achieved by genetically engineering cells to lack functional P53 and overexpress c-MYC [2]. The need for these aberrations to be present before a bacterial-induced transformed cell becomes malignant indicates that additional factors besides the mere bacterial toxins are needed to drive carcinogenesis and to maintain cells in a malignant state.

Microbial dysbiosis resulting in major shifts in intestinal commensal bacteria often result in changes in CD4^+^ T lymphocyte populations *in vivo*, leading to an influx of Th17 cells, chronic inflammation, and eventually cancer [4–6]. Consequently, the inappropriate propagation of certain commensal species in the gut has been associated with mucosal inflammatory diseases and cancer development [3,7–10]. However, recent experiments investigating the relationships between enteric bacteria and cancer have exposed the ability of certain species of intestinal commensals to significantly influence tumor size and progression in mice [4,11]. In similar studies, pro-inflammatory Th17 and Th1 cells were at times found present along with anti-inflammatory Treg populations in the intestinal mucosa. This antitumor response was mediated by a balanced production of pro- and anti-inflammatory cytokines, resulting in a controlled threshold of mucosal immunity largely moderated by CD4^+^ T lymphocyte populations, through a dendritic cell-dependent pathway [12–17]. Certain species of commensals, such as *Bacteroides fragilis*, a gram-negative obligate anaerobe, whose enterotoxigenic strain stimulates colonic inflammation and enhances colonic tumor formation, can at times improve host antitumor response by contributing to immune homeostasis through the balancing of CD4^+^ Treg, Th1, and Th2 cell populations [18–20]. More recently, Viaud et al. [4] demonstrated that commensals *L. johnsonii and E. hirae* were able to polarize T cells into Th1 and Th17 cell phenotypes and elicited a strong antitumor response in mice treated with chemotherapy compared to germ-free control mice [4,21]. These findings provide new evidence that certain species of enteric commensals can help manage subcutaneous tumor development by calibrating mucosal and, in some instances, systemic thresholds of innate and adaptive immunity [19].

Numerous key advancements in microbiological studies now allow us to molecularly characterize and discern pathogenic microbes from beneficial gut microbial species such as commensals. These molecular distinctions are now being investigated for their contributing role in pathogenesis, particularly cancer of the mucosal tissue [1,2]. More recently, however, the influence that bacterial infections have on the development of certain cancers, such as gastric cancer and gall bladder cancer, has gained increasing attention [1,2,15–18]. Unlike viruses, bacteria do not integrate their DNA into the host genome [1,2]. Consequently, the role of bacteria in carcinogenesis involves many factors that are not host-cell specific, some of which include the chronic stimulation of inflammatory immune responses through a dramatic modulation of the host mucosal immune landscape *in vivo* [15]. In this article, we chose to explore the potential role of food-borne pathogens and enteric bacteria as immune regulatory agents with the potential to hinder primary cutaneous squamous cell carcinomas and adenocarcinomas of mammalian origin.

## 2. COMMENSALS REGULATE MUCOSAL IMMUNITY AND HOMEOSTASIS

Within mucosal tissue, a group of immune regulatory cells (Treg) and pro-inflammatory immune cells, T helper type 1 (Th1) and Th2, play symbiotic supervisory roles for each other, enabling the maintenance of a healthy microbiome and regulating the growth of gut microbial populations. This natural biological check and balance system is now being revealed as a potential key factor in the hindrance, but also sometimes the development, of several lines of epithelial cancers. In this article, we discuss the therapeutic potential of enteric commensal bacteria as a cancer management tool *in vivo*. In consideration of these studies, we believe that certain combinations of human intestinal commensal bacteria can be cultivated to impede tumor growth at local and distant tumor sites by modulating CD4+ T lymphocyte cell activation (Table 1).

## 3. FOODBORNE PATHOGENS INFLUENCE INFLAMMATION

Infections by food-borne pathogens remain a major cause of illness in people with immunodeficiency [30]. Food-borne pathogens such as *Salmonella* can dramatically influence the host’s immune landscape, leaving it in a state of chronic inflammation [1]. Therefore, when coupled with immune deficiencies, infections by foodborne pathogens may be fertile ground for cell transformation and cancer. Today, foodborne pathogens represent approximately 15% of all pathogenic ailments [31].

It is currently estimated that nearly 50 million food-borne infections occur each year, and pathogenic infections that lead to chronic inflammation are responsible for over 15–20% of all cancers worldwide, with foodborne pathogens making a large contribution of these cancer-causing infections [16,17,32]. Among the best documented of these is the causal relationship between *Helicobacter pylori* and stomach cancer. Studies describing *H. pylori*-associated health benefits and disease-causing effects have consistently demonstrated that its colonization involves strong Th1 and Treg responses [18,33]. The implication of these findings is that exogenously driven Th1 responses may discourage further upregulation of local Th1 responses by the host, thereby inadvertently preventing excessive gastric inflammation and gastroduodenal disease. Thus balancing these *H. pylori*-mediated Th1 responses may be a promising approach to better calibrate these pathogen’s health benefits [18].

**Fig. 1.**
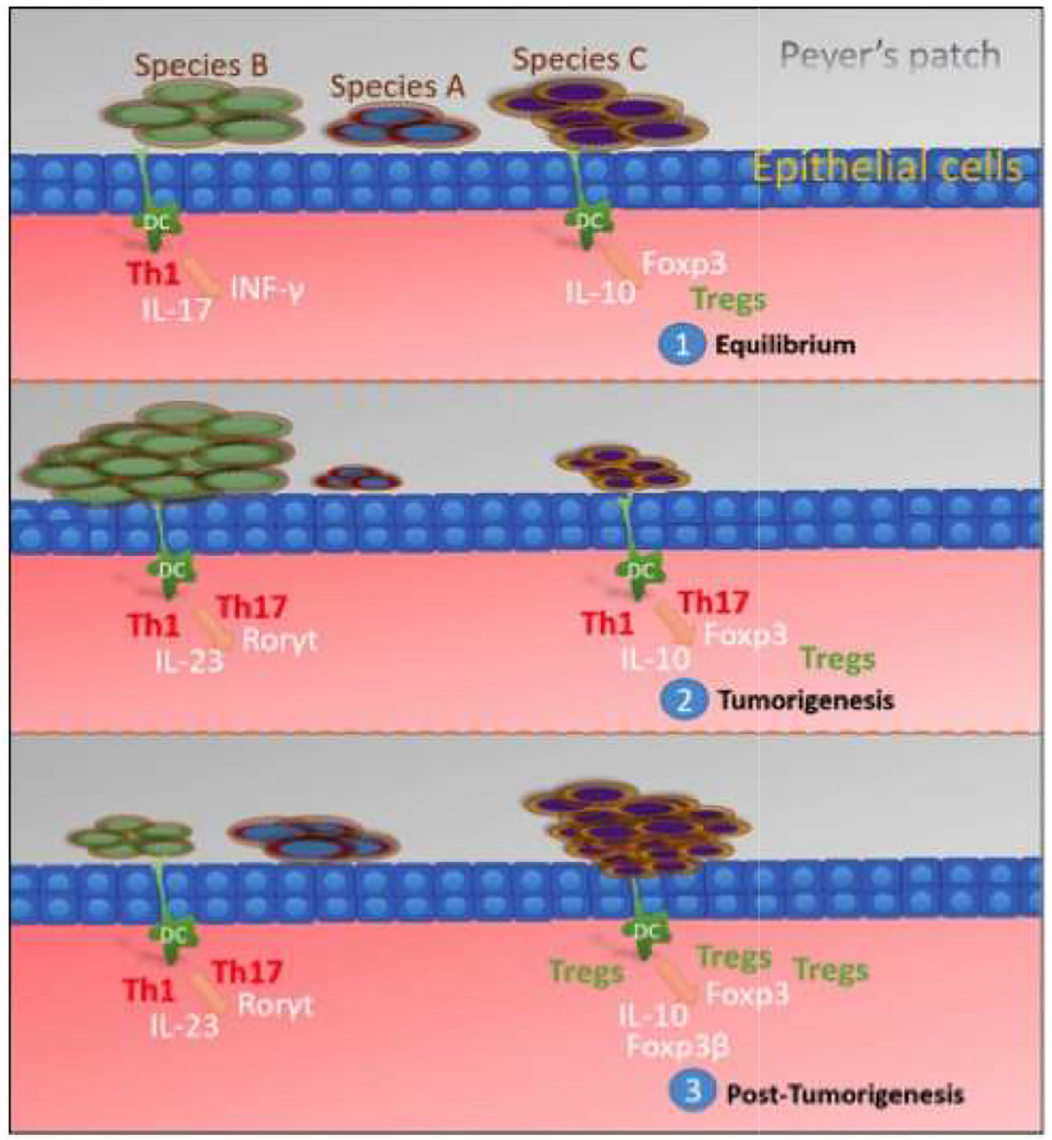
Schematic depicting the combinatorial role of foodborne pathogens and commensals on mucosal immunity homeostasis (1) The balanced presence of 3 bacterial species influences dendritic cell production, leading to the moderate presence of Th1 and Treg cells through the induction of INF-y and Foxp3 respectively. (2) Reduction of one or more species can lead to imbalance and promote tumorigenesis. (3) Signals induced during tumorigenesis can reshape the gut microbiome and stimulate the presence of pre-implanted anti-inflammatory Treg cells

On the other hand, the immune-protective effect of many strains of food-based bacteria is also well known. And certain groups of bacteria found in food have been demonstrated to boost or positively influence immune response [32]. Given these observations, it is likely that such strains can also have a preventative or perhaps even inhibitory effect against cancer of the digestive system, notably the gut. This is likely achieved by attenuating the presence of other groups of inflammatory bacteria in the gut (Fig. 1).

### 3.1 Bystander Effect

Studies such as that of Hansen et al. [33] have contributed significantly to the understanding of the bystander effect and the communal influence that microbial species exhibit on neighboring microbial species. This has led to the discovery that low levels of gastric Tregs are linked to an increased risk of peptic ulceration [15,18,33]. Despite the several links between gut bacteria and inflammatory diseases, the relationship between intestinal bacteria and human disease is highly contextual. Enteric bacteria can exist at different points between mutualism and pathogenicity depending on the immune and microbiological landscape of the host [34,35].

The majority of studies exploring relationships between bacteria and cancer emphasize immune changes taking place after bacteria inoculation in the host, but the microbiological context is often overlooked. Such studies are typically performed using a single murine intestinal bacteria species (i.e., a species that originates from mice), thus negating the combinatorial effects of enteric microbial species. Furthermore, the use of murine intestinal bacteria also undervalues the biological and genetic differences between the murine microbiome and that of humans [15,22]. To address this disparity, Faith et al. [22] developed procedures for generating germ-free mice through embryo transfer that also permit transplantation of human fecal microbiota intergenerationally, enabling researchers to study mice containing a “humanized” gut microbiome, and thus making it possible to conduct complex investigations heretofore impractical. Such humanized models have considerably expanded our abilities to conduct stage-specific studies involving metabolic and signaling pathways *in vivo*. More recently, these tools have enabled investigators to thoroughly characterize effector strains found in host microbiota, and permitted them to better define their influences on host immune regulation [36].

The shaping of human mucosal immunity depends highly on the presence of unique groups of enteric bacteria species known as “keystone species,” specific microbial species which have strong inhibitory or stimulatory effects on neighboring bacteria. Consequently these species can strongly influence and regulate mucosal immune response [19]. Similar species, such as *Enterococcus faecalis* and *Bacteroides fragilis*, have been described as putative contributors to immune homeostasis, yet their roles in immune homeostasis fluctuate depending on the enteric environment. Typically, commensal transition to commensal transition to a pathogen is favored to occur during chronic stimulation of pro-inflammatory cytokines (small cell signaling proteins that induce inflammation) and proliferation of the Th1 and Th17 T cell populations. Remarkably, further studies have demonstrated that certain species of gut commensals, such as *Lactobacillus johnsonii* and *Enterococcus hirae*, can polarize T cells into protective Th1 and Th17 cell phenotypes. These phenotypes elicit a strong antitumor response in chemotherapy-treated mice as compared with germ-free controls.

Analogous experiments also demonstrated that protective Th1 and Th17 responses were compromised severely in the absence of similar groups of commensal species [4,11,22]. These findings reveal that, depending on the microbial context, pro-inflammatory T-cell phenotypes could be beneficial against tumor growth, highlighting the pressing need for combinatorial microbial studies to determine consortia of enteric commensals that are beneficial against cancer development and tumor growth. Several of these studies were conducted by transplanting intact uncultured microbiota from human donors into germ-free mice. The culture collection was then randomly divided into groups and the modulatory effects on T cell regulation were monitored. This approach provided a more direct way to assess the modulatory effects of T cells on human microbiota by making use of human fecal content that harbored enteric microbiota from human subjects [4,11,22]. This experimental design is an amelioration of previous studies that have limited themselves to the murine microbiome. Yet studies using humanized-microbiome mice still have a prevailing drawback, since they continue to perform the immune assessment in non-humanized mice strains that possess a significantly different immune profile than that of humans. One approach or solution to this could be transplantation of intact uncultured human microbiota into a humanized mouse model with mucosal immune regulatory functions mimicking those of humans.

### 3.2 CD4+ T Lymphocytes Regulate Mucosal Immunity

Studies by Faith and colleagues, using a collection of bacteria in gnotobiotic mice, found that strains of the *Bacteroides* species and the broad *Bacteroidales* phylum were able to stimulate colonic Foxp3+ Treg cells among CD4^+^ T cells [22]. In their experiments, commensals *B. intestinalis* and *E. coli* were able to induce a significant increase in colonic Tregs, while the normal intestinal microbiota negative control, *Collinsella aerofaciens*, could not. These responses seemed to be greatly influenced by host diet, mostly composed of casein and starch, indicating that microbial metabolic byproducts could play an immune stimulatory role in the gut mucosa. These groups of commensals have been found to alter immunity not only in local tissue, but also in tissue sites distant from the gut [20,22,37]. These researchers identify enteric microbiota as key players in the shaping of immune signaling at the mucosal and systemic level. Analysis of splenocytes (a branch of immune cells that originates from the spleen) demonstrated that populations of CD4^+^ T cells were lower in germ-free mice compared to conventionally colonized mice, while the proportions of other lymphocyte populations, such as CD8^+^ T cells and B cells, were unchanged regardless of the presence of intestinal bacterial flora in the mice [22,38,39]. These findings articulate the significance of CD4^+^ T lymphocyte populations in enteric-mediated immune response and are supportive evidence that intestinal bacteria modulate mucosal and systemic immunity primarily through a CD4^+^ T lymphocyte-dependent pathway.

### 3.3 Intestinal Commensals Drive Antitumor Response

Certain enteric commensal species, such as the commensal bacterium *Lactobacillus plantarum*, have been found to reduce intestinal inflammation through the induction of protective interlukin-10 (IL-10), and are able to protect the host against inflammation-based mucosal diseases, such as inflammatory bowel disease (IBD) and perhaps cancer [3,40]. In addition, other species, such as *E. hirae*, have been demonstrated to be able to direct pro-inflammatory T cells to elicit strong antitumor responses [4,11,41]. Further investigation of these dynamics has revealed an increased survival rate in cancer-bearing mice that had been inoculated with a bacteria-derived product such as lipopolysaccharide (LPS) [7,11,42]. In these mice, LPS was able to improve host antigenic memory, eradicate tumors, and increase survival when compared to the control group [11,42].

The antitumorigenic abilities of microbial products, such as LPS, were equally explored in *Pseudomonas aeruginosa* by testing its signal molecule (O-DDHSL) on pancreatic carcinoma cells, where it significantly reduced pancreatic carcinoma cell mobility and viability. In their experiments, different concentrations of O-DDHSL were used on ductal epithelial cell lines (HPDE) and prostate cancer cells (Panc-1). Cell viability was then determined in both lines between 24 and 48 hr after treatment. The findings revealed that, compared to the control, treatment of cells with O-DDHSL concentrations between 25–300 μM resulted in a significant decrease in cell viability [43]. Table 2 summarizes the food borne and commensals with potential roles in chronic mucosal inflammation.

### 3.4 Commensals Play Functionally Contextual Roles in Cancer Development

Although many groups of intestinal microbiota have a mutualistic (i.e., coexisting without becoming pathogenic) relationship with their host, certain species can exist at different points between mutualism and pathogenicity [35]. Consequently, how commensal bacteria behave in the gut is highly contextual, with the same microbe becoming commensal or parasitic depending on the immune and microbiological landscape of the host [19]. Commensals that cause a deficiency in T-bet and Tregs (i.e., immunosuppressive T regulatory cells) usually induce pro-inflammatory Th17 cells, chronic inflammation, and eventually cancer. However, at low doses, endotoxins and commensal-associated molecular patterns (CAMPS) could potentially mediate moderate levels of inflammatory response that impede cancer cell growth and retard tumor progression in mice [7,58–60].

*Helicobacter pylori*, the etiological agent of stomach cancer, for instance, has been associated with protective properties against other types of cancers, such as esophageal adenocarcinoma, by preventing pan-gastric inflammation and reflux esophagitis in human hosts [18,33]. Such findings have redefined scientists’ understanding of microbial commensalism, and are consistent with the premise that combining bacterial species that induce anti-inflammatory response with those that regulate levels of protective pro-inflammatory signals can mediate a favorable antitumor environment that considerably impedes cancer development and tumor growth [22]. Various chemokins revealed their influential effects on foodborne pathogenic bacteria as summarized in Table 3.

## 4. BENEFITS AND LIMITATIONS

The experiments described in this article aim to illustrate the combined effects of enteric bacteria on systemic and local inflammatory response. Given the bacterial properties described by many laboratories, we expect that the combination of *Enterococcus hirae, Bacteroides fragilis*, and *Escherichia coli* will elicit the strongest response against tumor progression, as species within this group robustly stimulate Treg cell proliferation and induce high levels of protective anti-tumoral Th1 and Th17 cells. This can also be expected from less studied commensal species such as *Alistipes shahii, and Faecalibacterium prausnitzii* which have been demonstrated to be present in high numbers during mammalian tumor regression [4,11]. Although bacteria species could likely form *stable communities in rodents*, it is possible that some of these species will not survive when introduced to a humanized mouse model or when combined with human ATTC gut strains, highlighting the limitations of this approach. One of the ways to overcome this would be to maintain mice on a specific diet containing desired species of colonizing bacteria.

We expect that inoculation of mice, prior to tumor growth, with commensals able to maintain Foxp3+ Treg cell proliferation and induce moderate levels of inflammatory signals will mediate an antitumorigenic mucosal environment that considerably impedes tumor growth. We anticipate that once tumor growth is detected, moderate levels of inflammatory signals will likely be stimulated to create a toxic environment for tumor cells, while Tregs and Th2 cells will keep this pro-inflammatory response acute and manageable. Certain groups of cytokines in the serum or intestinal lavage may not be detected at the serum or peritoneal levels. For this purpose, the use of reconfigurable microfluidics combined with antibody microarrays for enhanced detection of T-cell-secreted cytokines may prove very beneficial.

## 5. CONCLUSION

Many studies have demonstrated that commensal-mediated antitumor response can be influenced by unique groups of food-borne pathogens and intestinal murine bacteria. The combined effects of enteric bacteria may have systemic and local inflammatory responses, in addition to cancer development and tumor growth. The proposed concepts exceed the mere investigation of mono-associations between bacteria and tumor regression, however emphasize the “bystander effect,” *i.e*. combined role of unique groups of gut commensal bacteria in host antitumor response [11,19]. Moreover, unlike previous studies, this article centers on the use of mice engrafted with a humanized mucosal immune profile as a reliable molecular tool for the investigation of microbial studies in a model system that mimics the human immune profile. With the pioneering advances in gnotobiotic biology, such as that of Faith et al. [22], it is fitting to speculate that inoculation of mice (prior to tumor growth) with commensals that are known to induce proliferation of anti-inflammatory cells, such as Foxp3^+^ Treg, and commensals able to moderate levels of inflammatory signals will create an antitumorigenic mucosal environment, which will considerably impede tumor growth. It could be anticipated that once tumor growth is detected, moderate levels of inflammatory signals are likely to be stimulated to create a toxic environment for tumor cells, while Treg cells and Th2 cells will be able to keep this pro-inflammatory response acute and manageable. These major shifts in intestinal commensal bacteria often result in systemic changes that can have whole-tissue antitumorigenic responses. Coupled with immunotherapy, these approaches may prove efficiency in presenting and eliminating dysplastic cells. Future research challenges would include identifying specific microbial candidates that could be maintained *in vivo* at sub-potent levels and administered to patients to cure or manage cancers. The challenges of this novel treatment and cancer management methodology are likely to require further thorough experimentation to help clearly define beneficial microbial combinations and their respective curative modes of action. Indeed, the modulation of commensal microbiota for the stimulation of immune response against cancer appears to be exceptionally promising.

## Figures and Tables

**Table 1.** Immune cells influenced by commensals and foodborne pathogens

Cell type	Food-borne pathogens	Regulatory role	Effect on immunity	Source
T and B lymphocytes	*Salmonella spp, Campylobacter*	Recognition of pathogenic invaders.	Depletion leads to increased susceptibility to food-borne bacteria infection	15
CD4^+^ T lymphocytes	*Enterococcus faecalis*	Orchestration of adaptive immune response in the gut	Presence controls threshold of mucosal immunity	12–14
Th17 cells	*Salmonella* spp., *Escherichia* coli, *L. johnsonii* and *E. hirae*	Orchestration of the mucosal defense against pathogens.	Characterized by their expression of the pro- inflammatory cytokine interleukin-17.	4–6
Treg	*B. intestinalis*	Prevention of colonic inflammation	Characterized by their expression of the anti-inflammatory cytokine	22, 23
Th1	*Bacteroides fragilis, L. johnsonii and E. hirae*	Regulation of Th1/Th2 equilibrium	Involved in pro-inflammatory response	4, 24–27
Th2	*Bacteroides fragilis*	Induces secretion of Th2 type cytokines	Leads to higher production of IgE	26–28
Macrophage	*Salmonella* spp.	Recognition of pathogen-associated molecular patterns	Surveillance of gut mucosa	29

**Table 2.** Foodborne and commensal bacteria with potential roles in chronic mucosal inflammation

Species	Regulatory role	Source
*Salmonella* spp.	Induction of pro-inflammatory cytokines through secretion of carcinogenic endotoxins and cytolethal distending toxins (CDT)	1, 2, 44
*Escherichia coli*	Induction of pro-inflammatory cytokines through secretion of carcinogenic endotoxins and CDT	1, 44, 45
*Bacteroides fragilis*	Stimulation of colonic inflammation and enhancement of colonic tumor formation	13, 18–20
*Lactobacillus johnsonii*	Polarization of T cells into Th1 and Th17 cell phenotypes	4, 19
*Enterococcus hirae*	Polarization of T cells into Th1 and Th17 cell phenotypes	4, 11, 41
*Bacteroides intestinalis*	Increase in colonic Tregs	4, 11, 41, 46
*Lactobacillus plantarum*	Influential role on intestinal and systemic immunity	3, 40
*Pseudomonas aeruginosa*	Reduction of pancreatic carcinoma cell mobility and viability	43
*Helicobacter pylori*	Protective anti-tumorigenic properties	18, 33

**Table 3.** Chemokines with influential effects on foodborne cancer-modifying bacteria

Cytokine	Cancer-modifying bacteria	Regulatory role	Immune influence	Source
IL-10	*Lactobacillus plantarum*	Protection against inflammation-based mucosal diseases and cancer	Reduction of mucosal inflammation	3, 40
IL-4	*Escherichia coli*	Reduction of mucosal inflammation	Reduction of Th2 cells	47
IL-6	*Lactobacillus* spp	Balance of mucosal homeostasis	Regulation of inflammatory and non-inflammatory signals in the small and large intestine	48
IL-10	*Campylobacter jejuni*	Anti-inflammatory regulation	Contextual macrophage activation	29, 49
TGF-β	*Escherichia coli* and *Helicobacter pylori*	Immune homeostasis	Promoting regulatory T cell differentiation	50
IFNy	*Clostridium difficile* and *Cryptosporidum parvum*	Anti-inflammatory regulation	Essential mediator of anti-inflammatory immune response	51
IL-5	*Escherichia coli*	Pro- inflammatory response	Associated with a shift of the Th1/Th2 balance	28
IL-13	*Helicobacter pylori*	Pro- inflammatory response	Released by signals from an injured or inflamed epithelium	52
IL-17	*Salmonella* spp. and *Escherichia coli*	Pro- inflammatory response	Essential for inflammation mucosal immune response	5
IL-17A,	*Helicobacter pylori* and *Bacteroides intestinalis*	Pro- inflammatory response	Permeability and maintenance of mucosal barrier integrity	53
IL-17F	*Salmonella* spp. and *Escherichia* coli	Pro- inflammatory response	Mediate pro-inflammatory responses	54
IL-21	*Salmonella* spp. and *Escherichia coli Campylobacter* spp.	Pro- inflammatory response	Regulation of effector T cells in the gut	55
IL-23	*Helicobacter pylori* and *Bacteroides intestinalis*	Pro- inflammatory response	Master regulation of gut mucosal immunity	56
IL-27	*Helicobacter pylori*	Pro- inflammatory response	Control of intestinal T cell pool homeostasis and moderation of intestinal inflammatory response	57
